# Integrating blockchain and ZK-ROLLUP for efficient healthcare data privacy protection system via IPFS

**DOI:** 10.1038/s41598-024-62292-9

**Published:** 2024-05-23

**Authors:** Shengchen Ma, Xing Zhang

**Affiliations:** 1 Liaoning University of Technology, Jinzhou, China; 2 Key Laboratory of Security for Network and Data in Industrial Internet of Liaoning Province, Jinzhou, 121000 China

**Keywords:** Blockchain, EMR, IPFS, Zero-knowledge proofs, Engineering, Electrical and electronic engineering

## Abstract

With the rapid development of modern medical technology and the dramatic increase in the amount of medical data, traditional centralized medical information management is facing many challenges. In recent years blockchain, which is a peer-to-peer distributed database, has been increasingly accepted and adopted by different industries and use cases. Key areas of healthcare blockchain applications include electronic medical record (EMR) management, medical device supply chain management, remote condition monitoring, insurance claims and personal health data (PHD) management, among others. Even so, there are a number of challenges in applying blockchain concepts to healthcare and its data, including interoperability, data security privacy, scalability, TPS and so on. While these challenges may hinder the development of blockchain in healthcare scenarios, they can be improved with existing technologies In this paper, we propose a blockchain-based healthcare operations management framework that is combined with the Interplanetary File System (IPFS) for managing EMRs, protects data privacy through a distributed approach while ensuring that this medical ledger is tamper-proof. Doctors act as full nodes, patients can participate in network maintenance either as light nodes or as full nodes, and the hospital acts as the endpoint database of data, i.e., the IPFS node, which saves the arithmetic power of nodes and allows the data stored in the hospitals and departments to be shared with the other organizations that have uploaded the data. Therefore, the integration of blockchain and zero-knowledge proof proposed in this paper helps to protect data privacy and is efficient, better scalable, and more throughput.

## Introduction

After decades of development, healthcare management systems^1^ have hitched a ride on the great wave of Internet development and have made tremendous progress in various technologies, including front- and back-end. Naturally, this has facilitated the development of traditional record-keeping methods for patient management, diagnosis, treatment, equipment management, and so on. However, electronic medical records or electronic personal healthcare^2^ data is the trend, the maintenance of EMR^3^, EPHD is one of the important tasks of hospitals at this stage, not only its lies in the protection of patient privacy aspects, but also in the academic exchanges of hospitals with each other, the ultimate beneficiaries of the data flow is still the patients themselves, so it is necessary to solve the problem of data silos as an important issue in order to provide patients with quality services, accurate diagnosis and treatment. In summary, the contributions are summarized as follows:

- This article combines traditional blockchain with IPFS network and proposes a scalable medical data privacy protection framework, which can ensure data security while enhancing data utilization.
- This article focuses on data and makes all processes serve data. Although the power of doctors is appropriately increased, submitting smart contracts for identity verification ensures security. At the same time, standardizing the medical data process in the model eliminates the problem of data silos in academia. We also found that this model produces better results, especially in relatively closed scenes.
- Under the same gas, our method outperforms other methods in terms of gas consumption and speed. Multiple experiments have shown that the proposed method has better applicability and lower consumption compared to cloud services and other scaling methods.

### Traditional systems

Traditional healthcare systems^4^ are highly centralized with multiple data privacy and security risk factors. It may act as a single point of failure paving the way for potential losses, data, tampering, alteration of records, etc. due to which the data is centralized. For organizations, a patient may need to repeat the same test when he goes to another hospital leading to financial loss. Policies and data standards followed by different organizations vary widely, hampering interoperability between organizations. Data breaches have been documented in the past in various healthcare organizations. This requires better healthcare data infrastructure. Some of the studies conducted have concluded that healthcare systems have become active targets of cyber attacks^5^ by malicious adversaries. The presence of healthcare data in a centralized client–server architecture within an organization makes it very challenging to collaborate with data across various providers.

### Cloud based

The centralization of data in the traditional model suffers from accessibility, scalability, privacy and security issues; it also acts as a single point of failure with limited recovery options. Cloud computing^6^ comes to the rescue and allows healthcare networks to store their data in the cloud infrastructure, which solves the accessibility, scalability and some privacy and security-related constraints. Patients and organizations such as hospitals retrieve patients’ health data remotely through cloud-based servers. Cloud-based systems may offer several advantages over traditional healthcare systems; however, they still have potential risks due to centralization. Unauthorized agencies may have access to cloud-based systems, which could affect the privacy and security of stored data. Another aspect is that cloud-based approaches fully trust third-party^7^ cloud service providers, allowing them to manage access control permissions for patient-generated health data. Healthcare data is very sensitive and cloud providers may utilize it for commercial gain without patient’s consent. The centralized nature of the cloud infrastructure could lead to potential bias and corruption when disputes arise between stakeholders such as patients, healthcare providers, and research organizations.

### Necessity of healthcare requiring blockchain

As the healthcare industry continues to grow, the volume and importance of healthcare data continues to increase. However, the traditional centralized storage method has a high risk of leakage. Blockchain technology^8^ can achieve the protection of medical data through decentralization. First, data backup can be established using blockchain technology, avoiding the risk of data loss due to the failure of a single node. At the same time, due to the distributed nature of blockchain, data can be stored on multiple nodes, increasing the security of data. Secondly, blockchain technology can realize data encryption. By using advanced encryption algorithms^9^, medical data can be encrypted and stored to ensure that only authorized people can access and manipulate the data. Blockchain technology combines advanced encryption algorithms to encrypt and store medical data. Through the setting of public and private keys, only authorized people can access and manipulate the data. This mechanism effectively protects the privacy and rights of patients and avoids data leakage and misuse. At the same time, it also sets a higher standard of trust for healthcare organizations, providing a strong guarantee for the healthy development of the healthcare industry and the safety of patients’ data. In addition, blockchain’s smart contracts^10^ can be used to restrict data access and operating privileges, avoiding unauthorized access and data leakage. As the data on the blockchain is not tamperable, data can be avoided from being maliciously modified or deleted. Meanwhile, using smart contracts on blockchain, automatic verification^11^, sharing and exchange of data can be realized through set rules, which improves data interoperability between different healthcare institutions and ensures that only operations that comply with the rules can be executed, thus protecting the authenticity and integrity of data. In conclusion, it is of great significance to use blockchain technology to address the risk of leakage of centralized medical data. In addition, smart contracts can be used to automate the payment and sharing of data, bringing more business value to the healthcare industry. These features provide a more convenient way for collaboration between medical institutions, which is conducive to the optimal allocation of medical resources and the enhancement of the patient experience. In short, the security and reliability of medical data can be greatly improved by establishing data backup, realizing data encryption and ensuring data integrity. In the future, with the continuous progress of technology and the expansion of application scenarios, it is believed that blockchain technology will play a greater role in the medical field.

## Blockchain fundamentals in healthcare sector

### Merkle tree

Merkle Tree (Merkle Tree) is a data structure based on blockchain technology that allows us to group large amounts of data into a tree structure, making it possible to verify the integrity of the data without having to download the entire dataset in its entirety. Merkle trees work by grouping and hashing raw data and then recursively linking these hashes together to form a tree structure. Each node contains the hash values of its children, and each node’s hash value is derived by hashing its children. In this way, the hash value of the root node represents the integrity of the entire data set. In a Merkel tree, any change in the data results in a change in its hash value, so the integrity of the data can be verified by simply comparing the hash values of the root nodes of two Merkel trees^12^. If the root node hashes of the two Merkel trees are the same, then they represent the same data set. If different, then at least one of the datasets is incomplete. Merkle trees have a wide range of applications in blockchain technology, such as in cryptocurrencies like Bitcoin^13^ and Ether, where they are used to verify the legitimacy and security of transactions. Also, Merkle trees can be used for distributed storage and data integrity verification, among other things.

### Time stamping

Time stamping^14^ technology is an important mechanism used to ensure data integrity and security. It ensures the immutability and authenticity of data by adding a time stamp to the data and associating the data with a specific time. In this article, we will introduce timestamping technology in blockchain in detail. The main application area of timestamping technology is to record and verify the existence and time of occurrence of data. It can be used in areas such as copyright protection of digital files, precise recording of the time of occurrence of events, digital twins, etc. Timestamping technology is particularly important in blockchain technology because the blockchain itself is a distributed ledger that needs to ensure the authenticity and tamperability of all data. Timestamping technology works by binding data to time to form a timestamp. This timestamp is generated by multiple nodes of the blockchain network, each of which has a local clock and is involved in the timestamp generation process. Specifically, when a transaction is added to the blockchain, in addition to the transaction itself, it will include information such as the hash value of the previous block, the timestamp, and a random number. Together, this information is packaged into a new block and added to the blockchain. Since each node’s clock is independent, timestamping ensures that all nodes have recorded this transaction information at the same point in time. Timestamping technology has a wide range of applications. For example, in the field of copyright protection, timestamps can be used to mark the creation time and copyright attribution of digital files; in the field of healthcare, timestamps can be used to record the patient’s condition and the doctor’s diagnosis results to ensure the authenticity and tamper-proofness of the medical data; in the field of finance, timestamps can be used to record detailed information about the transaction to improve the security and transparency of financial transactions. The advantage of timestamping technology is that it can effectively ensure the integrity and security of data. Since timestamps can associate data with a specific time, they can prevent data from being tampered with or falsified. In addition, timestamps can be used to track the origin and destination of data, thus helping people to quickly locate problems and take appropriate measures.

### Consensus

POW (Proof of Work)^15^ is a common consensus algorithm that earns the right to add a new block by requiring participants to prove their workload by solving an intractable mathematical problem. In a blockchain network, when a node wants to add a new block to the chain, it needs to first verify that the transactions in the block are legitimate and that the block conforms to the rules of the blockchain network. If the node cannot verify this information, then it will be denied to add a new block to the chain. The node also needs to generate a new block by solving an intractable math problem and broadcast the block to other nodes. The other nodes will validate the block to ensure that it complies with the rules and protocols of the blockchain network. If the block passes the validation, then it will be added to the chain. In order to prevent malicious nodes from attacking the blockchain network, the POW consensus algorithm constantly increases the difficulty of the math problem^16^. This makes it necessary for malicious nodes to pay more time and computational resources to solve the math problem, thus increasing the cost and difficulty of the attack.

### Public and private keys

Asymmetric encryption plays a crucial role in blockchain technology. It provides security and trust mechanisms for the blockchain, allowing us to use digital assets for transactions and storage with peace of mind. Asymmetric cryptography is based on public and private keys^17^. The public key is publicly shareable and can be used by anyone to encrypt messages or verify digital signatures. The private key, on the other hand, is highly confidential, accessible only to the owner, and it can be used to decrypt messages or generate digital signatures^18^. In blockchain transactions, the use of public–private key pairs provides security for participants. When a participant wants to send an asset to another participant, he can use his private key to sign the transaction and broadcast the signed transaction to the entire network. The receiver can verify the legitimacy of the transaction using the broadcasted transaction and the sender’s public key. If the verification is successful, the transaction is considered reputable and the asset transfer is confirmed and added to the blockchain. Another application of asymmetric encryption in blockchain is to achieve anonymity and privacy protection. The nature of public and private keys allows participants to protect their privacy and identity. The use of public keys for transactions allows others to verify the legitimacy of the transaction, but there is no way to know who initiated the transaction. The confidentiality of the private key allows only the true owner to access and manipulate the asset.

## Related work

Nguyen et al.^19^ Technologies such as blockchain, sidechain, cloud platform, and IPFS are shining in various fields, especially in the IoT industry, where blockchain is deeply bound to it. The proposed framework utilizes distributed technologies such as blockchain, exploits the potential of sidechain, provides reliable access control through smart contracts and ensures system security. Its evaluation methodology highlights performance improvements to the blockchain, cuts down on network consumption, and greatly improves the security of the system. Meanwhile, the framework can be extended, but using third-party cloud services can lead to trust or mischief issues, and when the content service provider is not fully trusted, the data can be easily tampered with and the system can be in danger. In different scenarios, the use of blockchain to solve different problems, in this article, although the deployment of the system on cloud services is a centralized behavior, but all other indicators are more efficient and faster than the general decentralized system architecture^20^, then so in some of the areas where decentralization is not too important, can make a big difference^21^. This thesis provides a patient with a treatment log, which is trusted, while accessing it is uncomplicated, participants can make changes to the medical data, and privacy is ensured by utilizing smart contracts for participant interaction. Using blockchain technology, MedRec demonstrates how the principle of decentralization can be applied to large-scale data management in electronic medical record systems. But one of its drawbacks is that the component about miners is not very clear. Also the generalization of the framework is not yet well developed^22^. This article utilizes blockchain technology to deal with the problem of data management in electronic medical record healthcare systems. Patients can easily access the medical records of different hospitals through its framework, thus eliminating the data silo problem, and the interaction through blockchain can help the doctors of the hospitals to have first-hand data to understand the patient’s medical history during consultation. The proposed blockchain-based efficient privacy protection scheme utilizes access control protocols and encryption to ensure data security. Its feature of not uploading data to a third party increases security. However, this framework has a more important problem that needs to be solved urgently, that is, it doesn’t use smart contracts to process the data but directly uses the blockchain to solve it, which creates a problem that the TPS will be very low, and at the same time, the ability to process the data is very inefficient because the throughput of the framework with no off-chain storage is only about a dozen of transactions per second, so the speed is a hard hit.

This solution also uses the blockchain + IPFS architecture to process data, where the advantages are similar and the disadvantages are also similar. Again, the lack of integration of smart contracts leads to a low TPS^23^.

In this paper, blockchain technology is combined with an attribute-based access control model, which makes full use of blockchain technology to break the information silos in medical data, and safeguard the security and privacy of medical information^24^. In addition, the star file system is used for storage to alleviate the storage pressure of blockchain. The article also utilizes smart contracts to process data in specific intervals, but its IPFS is only an alternative to the on-chain space and another as a distributed database, which only alleviates the problem of small throughput and does not actually solve it, which is of course its focus to be researched in the future^25^. The paper proposes a user-centric healthcare data-sharing scheme for privacy-preserving machine learning, which implements encrypted data storage, blockchain-based data resource allocation, data authorization, and machine learning model training. It also designs an auditing mechanism to help users audit the data-sharing process. Compared with existing schemes, our proposed scheme ensures the privacy and security of user data, guarantees user data ownership, and achieves data exclusivity and non-visualization. Finally, the functionality of the solution is realized through simulation experiments, and the experimental results prove the feasibility and effectiveness of the solution. But throughput is also an urgent problem for this article^26^. It focuses on the blockchain-based medical data-sharing solution and proposes the B-SSMD system solution. By combining the three-chain model with IPFS technology and adopting the method of storing data on the chain and indexing paths of the chain, it realizes highly distributed data storage and solves the defects of centralized storage in traditional medical systems. In addition, using three-layer encryption and fine-grained access control, the P/MSISP data-sharing process is proposed to protect user privacy and improve data security. Finally, the security analysis proves that the paper’s scheme is secure, and simulation experiments demonstrate the feasibility. For further research work, one possible improvement is the transferability of the B-SMD system. How to use the triple-chain model to solve the data sharing problem not only in healthcare environment but also in other scenarios is a future work that can be explored. However, in this framework, its three-layer encryption and fine-grained access control are slightly redundant. The cost of more processes is high network consumption^27^. In the model proposed in this article, the access control mechanism is a good choice to reach the purpose of data sharing security, but the limitations of scalability and expansion mechanism lead to the low throughput of this model, and consequently the usability is not as high^28^. Unlike other articles, the framework in this article, gives the data owner to the patient, which has the advantage of opening access to its own data based on choice, but this breaks the rules of sharing. At the same time, its efficiency in terms of expandability, data availability, and security gets reduced to different degrees.

This paper proposes a holochain-based privacy-preserving secure communication scheme for distributed IoT healthcare applications, which utilizes the inherent autonomy of the holochain architecture and protocol^29^. Contrary to blockchain, holochain frees communication agents from any form of centralized control by running applications (hApps) entirely on the user’s end. As a result, there is no central point of failure. Comparative performance results and analysis show that the time and space complexity of the new chain framework is significantly reduced compared to competing blockchain solutions, suggesting the promise of real-world deployment of large-scale IoT healthcare systems. Although the problem of processing GAS in real-time has not been effectively addressed, the paper breaks with tradition and the efficiency of the newly proposed model is indeed improved^30^. The proposed method is based on the availability and accessibility characteristics of blockchain for distributed service response. The proposed method is based on the availability and accessibility characteristics of blockchain, which will be used for distributed service response. There has been a breakthrough in IoT, but the consensus mechanism has been a headache for non-arithmetic devices^31^. Disease prediction is also more effective than other deep learning models as the proposed model yields higher prediction accuracy. But fails to consider data protection issues and other attacks in lost data scenarios, though its combination with deep learning models is a highlight^32^. This framework discusses privacy issues in the rapid establishment of medical progress monitoring in decentralized communication systems in the medical Internet of Things. It adopts a hybrid hash design and integrates homomorphic encryption algorithms to support smart contracts with decentralized applicability. The proposed method has high mobility.

As shown in Table 1, the attributes and characteristics of some references are listed, from which it can be seen that the existing methods have some shortcomings in various indicators, but the control of access and distribution are basically necessary, and many of them increase the speed, but also lead to the ultimate problem in the blockchain expansion.

**Table 1:** Advantages and limitations of state-of-art schemes.

Research work	Access control	Extensibility	Lower network consumption	Decentralization	Used cloud	Smart Contract	High efficiency
^19^	√	√	√	×	√	×	√
^20^	×	×	√	×	×	×	√
^21^	√	×	√	√	×	√	×
^22^	√	×	√	√	×	×	×
^23^	√	×	√	√	×	×	√
^24^	√	×	√	√	×	√	√
^25^	×	×	×	√	×	×	×
^26^	√	×	×	√	×	×	×
^27^	√	×	√	√	×	×	×
^28^	√	×	√	√	×	×	×
^29^	×	×	√	√	×	×	√
^30^	√	×	√	√	×	×	√
^31^	×	×	×	×	√	×	×
^32^	√	×	√	√	×	√	√

**Figure 1:**
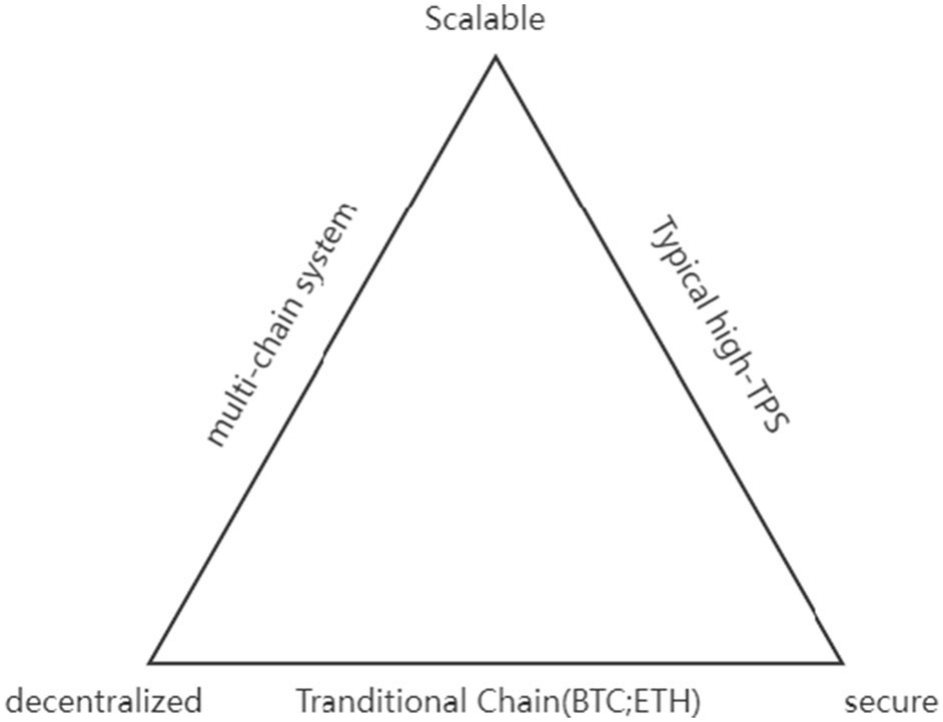
Scalability trilemma.

As shown in Fig. 1, the trilemma. it can be seen that if both decentralization and scalability are considered, security must not be guaranteed. In fact, all papers in related work guarantee security, but because they all have this commonality, they are not reflected in the table. Therefore, while ensuring security, scalability or decentralization must be abandoned to ensure the completeness of the other feature.

## Components of proposed decentralized storage

### IPFS

IPFS (InterPlanetary File System) is a peer-to-peer distributed file system designed to change the way files are stored and transferred on the Internet. Unlike traditional file systems, IPFS uses content addressing to identify and retrieve files, rather than being based on file paths. The basic concept of IPFS is to store files in chunks and generate unique fingerprints (hashes) for each file chunk. These fingerprints are used as addresses for the files rather than file paths based on the server and directory structure. This means that identical blocks of files will only be saved once, no matter how many users request access to them, thus increasing file-sharing efficiency and reliability. IPFS utilizes a distributed network model in which nodes communicate with each other and replicate data via a coherent protocol. Each node can store and transfer blocks of files without the need for a centralized server. When a node requests a file, it looks up the file’s blocks by hash value and fetches the required data. If a node is offline or unable to provide file blocks, IPFS automatically looks for other nodes to get the required data. Interplanetary File System (IPFS)^33^ is a sub-protocol package and project driven by Protocol Labs.IPFS is designed to improve the efficiency of the network and make it more decentralized and resilient.IPFS uses content-based addressing, where content is not addressed by its location but by its content.IPFS achieves this through its deduplication feature of storing and addressing data in a manner that efficient storage of data. With IPFS, files can be stored and shared in a decentralized manner, increasing the resistance to censorship of their content.IPFS can be deployed to build websites with a distributed web. It is used as a storage service that complements the blockchain by supporting different applications on top of IPFS. Since IPFS uses content-based addressing, it focuses primarily on immutable data. However, IPFS supports updatable addressing of content through the Integration of the Interplanetary Name System (IPNS).IPNS allows to link a name (a hash of a public key) to the content identifier of a file. IPNS entries are signed by a private key and can be arbitrarily (re)published to the web (default 4ℎ). Each peer maintains its own LRU cache of parsed entries (default 128 entries). IPNS entries have a specific lifetime, after which they are deleted from the cache (default 24ℎ). File updates can be achieved by changing the mapping of fixed names to content identifiers. Note that content identifiers are unique and file-specific. In addition, IPFS uses its own incentive layer, Filecoin^34^, to ensure the availability of files in the network. However, IPFS works independently of Filecoin and vice versa. This is a typical example of how cryptocurrencies can be integrated to incentivize peers.

### ZK and ZK-rollup

ZK Proof Using cryptography allows someone (the prover) to prove to someone else (the verifier) that the facts are 100% true, but without revealing any additional information beyond the statement of specific truth. That is, it can be kept secret and still be believed. For example, a ZK proof^35^can be used to prove the validity of an Ether transaction that sends a Token from Account A to Account B without revealing the Token balance of either account or the amount sent. The next is ZK-rollup. The principle is to compress and store the user state on the chain in a Merkel tree, and dedicate the changes of the user state to the chain, and at the same time, guarantee the correctness of the user state change process under the chain through the proof of ZK-snark/ It is costly to deal with the changes of the user state directly on the chain, but costly to utilize the smart contract to verify the correctness of a zero-knowledge proof of the PROOF, and the transfer information and the proof will also be submitted to the smart contract together. The transfer information and proof will also be submitted to the smart contract together to facilitate the checking of accounts.

#### SNARK

Simple Non-Interactive Knowledge Base (SNARK) allows to provide verifiers concise proofs, i.e., proofs that can be verified in logarithmic time. There have been many improvements to this type of scheme since the first practical protocol. In particular, Groth allows to provide fixed-size (less than 200 bytes) proofs that can be verified in constant time, regardless of the program to be proved. The main drawback of this scheme is that it relies on a trusted setup that must be performed for each different program, resulting in a common reference string^36^ (CRS), also known as the verifier and prover keys. The verifier key is sometimes different from the prover key, since precomputation can be performed for the verifier. The prover key is the same as the CRS. Even if SNARK is subject to a trusted setup, it is possible to divide the trusted setup into two parts: one part that depends on the program to be checked but has no trusted third party, and another part that is independent of the program and on which security is based. This independent part can consist of several participants, distributes security among them, and can be used for any program up to a certain number of operations. The result of this multiparty protocol is the same as the classical trusted setup, but trust is no longer based on a single participant. The idea is that security can be ensured if at least one participant is honest during the ceremony. Repeated use of completed rituals that include many well-known people helps build confidence in this model.

#### STARK

The Scalable Transparent Knowledge Base (STARK) differs from SNARK in the lack of a trusted setting. Scalability also implies that, in addition to being logarithmic in verification time, like SNARK, STARK must also be at most linear in proof time. Furthermore, STARK relies only on the properties of hash functions and Reed-Solomon codes, which makes them post-quantum (post-quantum cryptography aims to secure information against attackers equipped with quantum computers). However, STARK is the most recent proposal, and due to its proof length, more research is still needed to compete with SNARK in certain areas, and more precisely in blockchain.46267267.

## Proposed model

**Figure 2:**
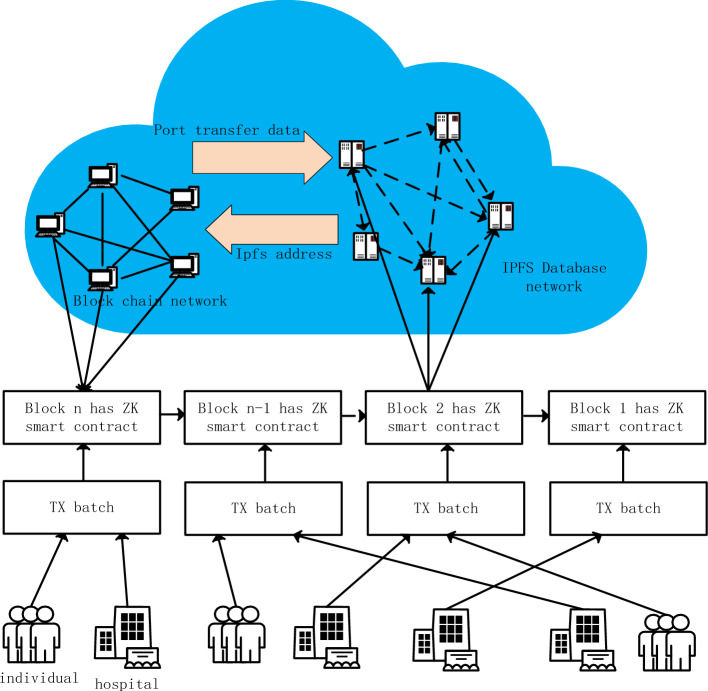
Framework overview and process.

The proposed model ensures that the rightful owner has the required access control over their health data, in our case the patient. Patients use cryptography applied within the system to protect their data from unauthorized access, misuse or fraud. Patients don’t need to know how cryptography works or how encryption is done. They can use the front-end system like any other web application and provide access to the physician/hospital entity when needed. When the access is over, they may revoke it. In this way, any user with basic computer knowledge can join this proposed network and protect their health data. All the cryptography, consensus mechanism, storage and retrieval is done within the system by design. Figure 2 shows the integration of blockchain with IPFS, while data generated by individuals or hospitals is given to TX Batch, and then this data (TX) is uploaded to a zero-knowledge proof smart contract, and the index of the aggregated information is placed on the main chain, with the specific details of the data being held by IPFS together with the smart contract (there is a high degree of freedom, there is no focus, and it doesn’t matter which one stores more or less).

### Overview

Figure 2 shows the integration of blockchain and IPFS, while providing personal or hospital-generated data to TX Batch, which is then uploaded to a zero-knowledge proof smart contract, and the index of aggregated information is placed on the main chain. The EMR generated by communication between patients and doctors is directly written into it as key-value pairs according to scientific research needs or all settings. Doctors directly upload the file to the IPFS network through command-line tools or customized client software to obtain the corresponding CID (content identifier). But it is connected by the main chain in the middle, and users call the smart contract deployed on the Ethereum main chain and provide it with the CID obtained from IPFS and any related metadata or transaction information. Smart contracts may include logic to verify the validity of CIDs, such as checking for existence in the IPFS network, or performing operations involving file ownership transfer. Afterward, relevant transactions are submitted to ZK Rollup through the Layer 2 interface, including CID, transaction details, and zero-knowledge proof (proof that the files have been successfully stored on IPFS). ZK Rollup batch processes these transactions and generates validity proofs, and then writes the summary results and proofs into the Ethereum main chain. The following is the classification and detailed division of labor of nodes in this process. (In Fig. 2, individuals and hospital can refer to individuals and organizations. In this article, individuals refer to patients and doctors, aiming to emphasize universality.)

### Doctor nodes

The doctor node is different from the hospital node in that when a patient visits the hospital, a non-contested electronic medical record is generated according to certain rules, which is uploaded by the doctor to ZK’s smart contract and signed with his own private key, and then transmitted to the main chain through the smart contract. When the doctor visits or performs other activities that require reading medical data, he or she has to upload a smart contract that merely verifies his or her identity to prove that he or she is not an evil node, thus ensuring the security of the data. And then a series of operations such as adding, deleting, changing and checking can be performed. Doctors must be full nodes, although the data does not belong to anyone, patients and doctors have the right to use it, but the patient nodes do not need to be deeply involved in the construction of the network, and need the doctor nodes to maintain the operation of the blockchain. In addition, the doctor node is a contract user, each doctor has to interact with various contracts, so it must be a contract user, although there is a generated public and private key, but the key account is not in the scope of consideration.

### Patient nodes

Half of the patient nodes are light nodes^37^ unless there are special requirements, they are necessarily light nodes because they join the network generally temporary, most people use this network for less than a few hours or more than a few days, and the depth of participation is not enough, so they can only carry out the release of their own medical data and modify the transaction according to the doctor’s recommendations. Of course, the generation of electronic medical records is also based on the medical recommendations of doctors. But this data belongs neither to the patient nor to the hospital; the data is just data, it just happens to be medical data. In addition, every patient is a contract user and interacts with contracts, especially ZK contracts. They are not full nodes and therefore only store block headers.

### Hospital nodes(IPFS)

The hospital node is different from the doctor node, its main function is to maintain the system operation, provide arithmetic power, so that the whole system has a strong robustness, so the hospital node is necessarily a full node. It is equivalent to a Server in C/S architecture, but in this system it is decentralized, no institution or unit can be the traditional centralized database, only the blockchain serves this job. After the data OR transaction is uploaded to the chain by the smart contract, the returned summary will exist in IPFS, which not only improves the uploading speed but also ensures the security of the summary taken back from the blockchain and placed in IPFS after hashing.

### ZK-nodes

Overall, the nodes that perform ZK are not a particular class of nodes, but rather nodes that have the ability and arithmetic power to perform ZK. Some of the patient nodes, all of the doctor nodes, and the hospital nodes are ZK nodes, in contrast to the patient nodes, which sometimes have too little depth into the system and variable time to complete ZK proofs in real-time. Individual contracts on the main chain are used to hold all the money and keep a concise cryptographic promise that points to the state of the "sidechain" (typically a Merkle tree of accounts, account balances, etc.)

### Data flow and process

The following is a description using pseudocode.
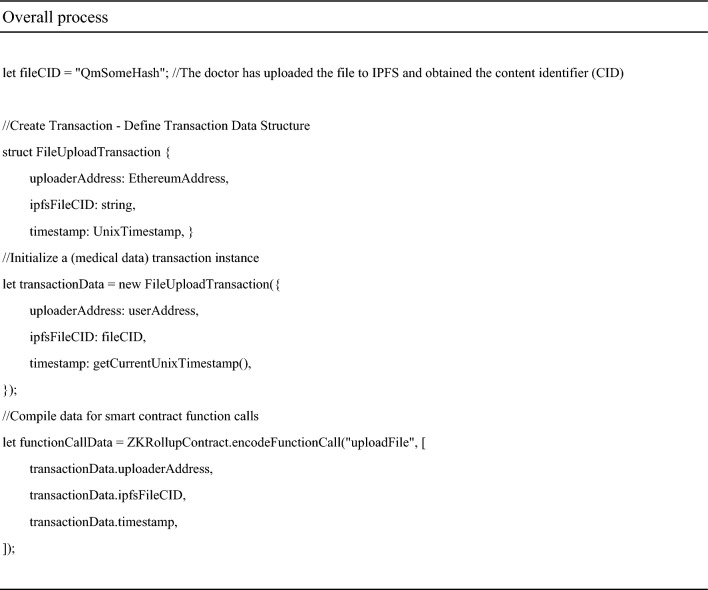


Submit transactions through the API or SDK provided by ZK Rollup await ZK-Rollup.submitTransaction (userWallet, functionCallData);
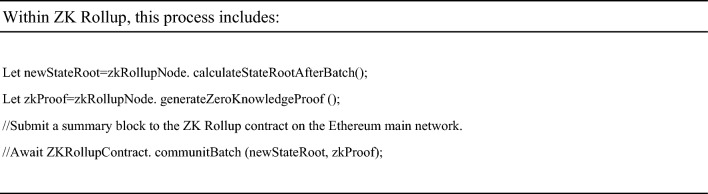


At this point, the smart contract on the Ethereum main chain will verify the proof and record the new state root. a) Add the transaction to the state machine of Layer 2 and wait for batch processing b) Generate a proof of validity for this transaction using a zero-knowledge proof algorithm.(Next is the logic of the ZK Rollup layer, and doctors usually do not directly participate in this step).When a batch of transactions is completed, the ZK Rollup node will calculate a new state root and proof.
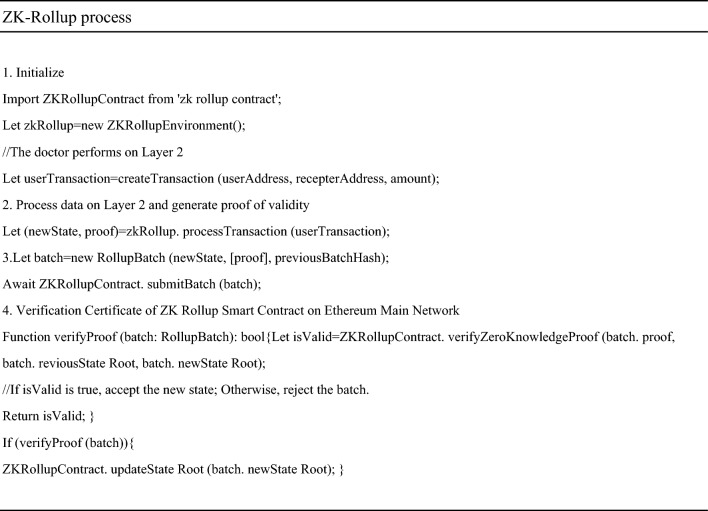


It includes applying transactions to the state tree and then generating proofs using the zk SNARK toolchain. Submit the compressed state and proof to the ZK Rollup smart contract on the Ethereum main network. ‘The submitBatch’ method is fictional and may involve multiple steps in practice, such as packaging the proof of multiple transactions into one batch, verifying batch integrity, and executing specific commit functions on the chain. After verification, the smart contract will update its stored latest status root. In this way, the Ethereum main chain only stores a small amount of validity proof data, while most of the calculation and transaction data is retained on Layer 2, thereby improving the scalability of the entire system.

**Figure 3:**
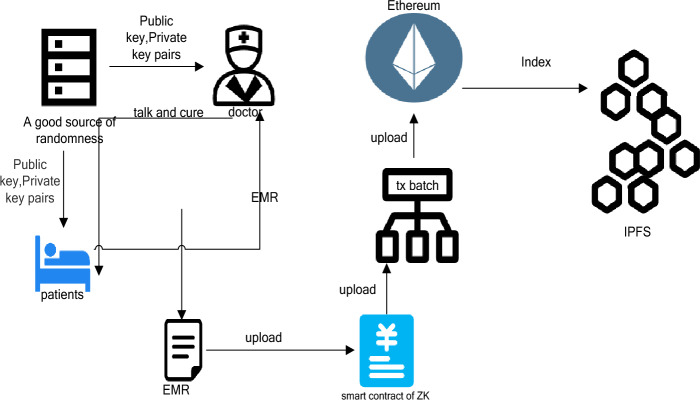
Data process details.

Figure 3 shows some specific processes. a good random source generates the public and private keys (RSA is used in the experiments and can be replaced depending on the actual.) Distributed to the patient and the doctor so that they each keep their own information, based on the visit information, the doctor determines the EMR, according to the order of uploading the data was submitted to the ZK Smart contract, after the contract processing, after queuing to the Ethernet main chain, at this time the information on the Ethernet is just the index (summary) of these data. Later, the index of these data can be placed in IPFS to further free up the contents of the chain and keep the data flow smooth.

**Figure 4:**
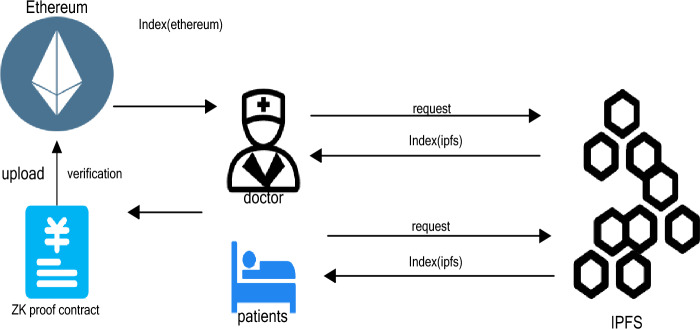
Patient and doctor node interaction with blockchain and IPFS network.

Figure 4 shows the process when either the patient or the doctor seeks medical data, by uploading their own proof of public–private key pairs to ensure their legitimacy and security, and then the request will be processed in the contract, and after submitting it to the main chain to generate an index, the index can be used to look for the required data in the framework.

## Performance evaluation

**Figure 5:**
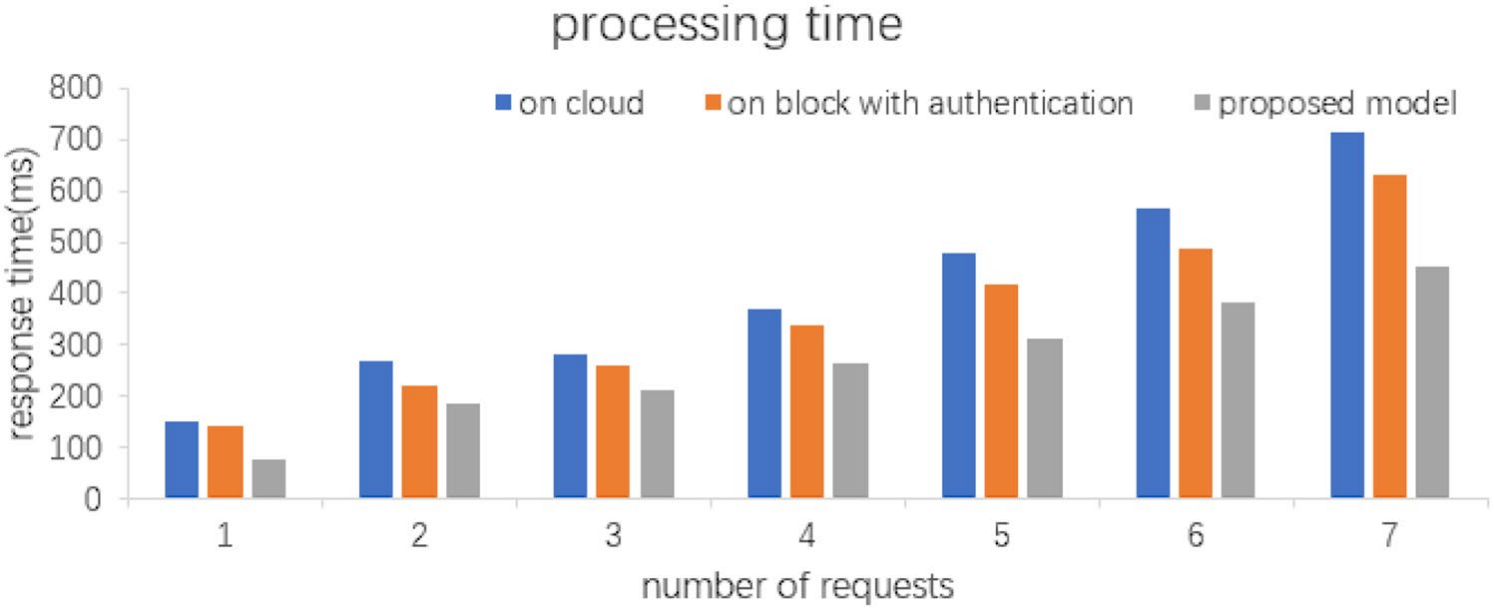
Comparison of processing time.

The metrics in Fig. 5 show the response time in cloud servers, other blockchains with authentication methods i.e., the model proposed in this paper for different numbers of REQUESTS, the response time varies due to the different locations of the cloud servers, in this paper Amazon cloud is used in the experiments.

**Figure 6:**
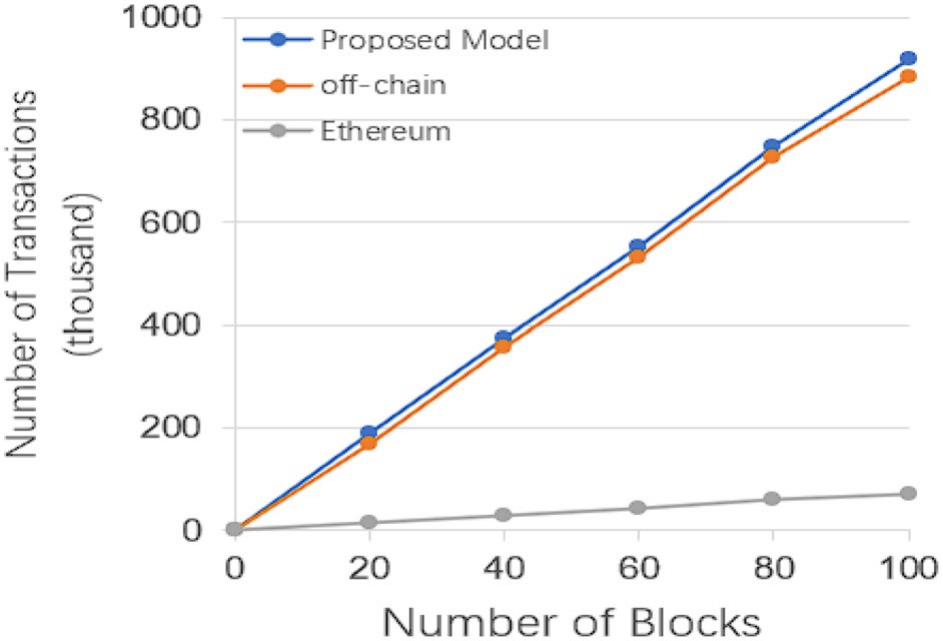
Number of transactions that can be handled by the number of blocks in different ways.

The metrics in Fig. 6 show the number of transactions the system can handle as the number of blocks grows, which doesn’t work well because Ether is simply too inefficient.

**Figure 7:**
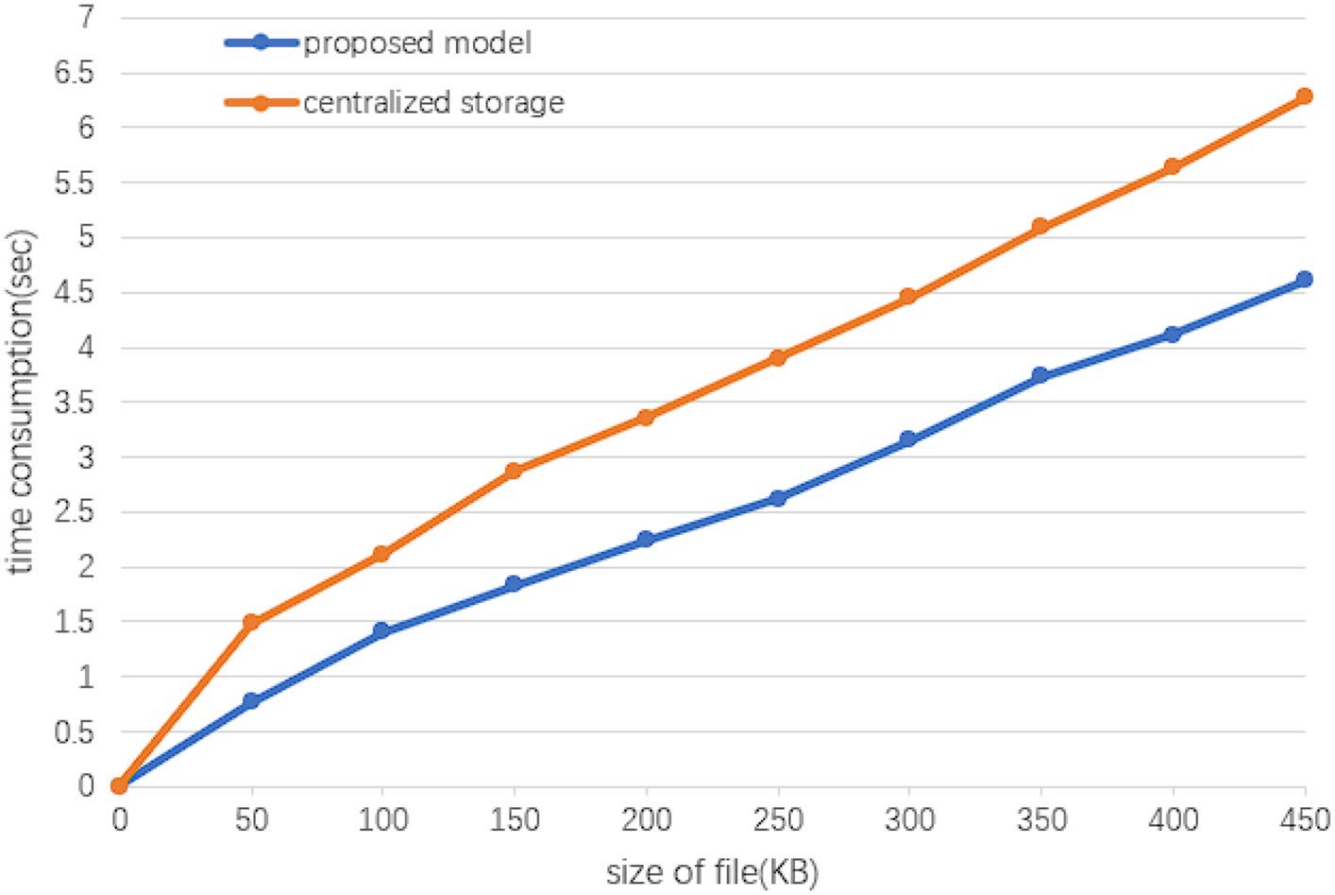
Response time in processing data.

Figure 7 demonstrates that the proposed model is less delayed and faster for a fixed amount of data, and the distributed storage is general blockchain integration IPFS, which is not too inefficient.

**Figure 8:**
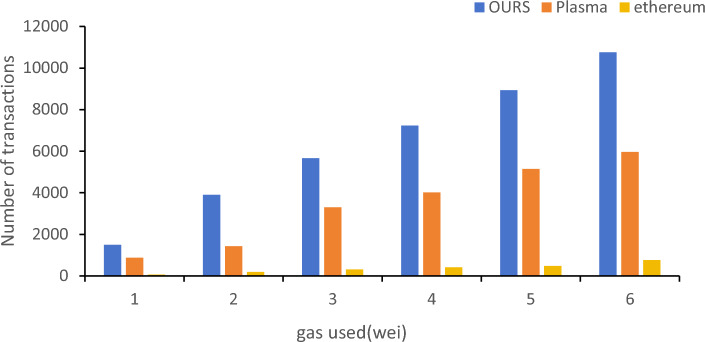
Comparison of Number of transactions under the same gas-used scenario.

Figure 8 shows the number of transactions corresponding to several data processing methods under the same gas fee. The proposed model mainly depends on the consumption of the verification part when submitting ZK proof. Plasma is another popular expansion method, and its fraud-proof time (usually at least one minute, which can be roughly understood as one block per minute) is a weakness in blockchain networks. Therefore, this article did not use the comparison with Plasma in terms of time. At the same time, through experiments and testing, the average gas consumption using Ethereum reached 22–25 wei, and the number of processed data was around 1500. At its peak, the gas consumption could even reach $100, which is approximately 0.04 ETH or 40 million wei.

We chose the evaluation indicators based on the following reasons: firstly, reaction time is one of the necessary indicators, which directly reflects the superiority of the model or module. At the same time, it is necessary to demonstrate the scalability of the model, so that the number of blocks and transaction volume can prove the volume that the model can accommodate. Secondly, the storage rate per unit file size is also of concern to us. Finally, uploading more data and transactions with the same gas fee is a crucial part of the entire blockchain research, and we have included it in our evaluation metrics.

## Conclusion and future work

In order to efficiently manage EHRs in a healthcare data network, a blockchain-based ZK-rollup framework is introduced and integrated with IPFS^33^, both of which are used for off-chain storage in the framework. The framework enables EHR storage to secure data in a decentralized architecture. Instead of being patient-centric or institution-centric, we let the system itself store the data and let the data belong to the data. The built-in design of the blockchain provides security and privacy through the effective application of cryptography. An additional layer of security is provided by storing encrypted data in IPFS storage. The encrypted EHRs are present in the smart contract and the data after the contract is uploaded to the chain is placed in IPFS, making the system lightweight yet efficient. The paper combines the scalability of IPFS and ZK-rollup to propose a network operation model with the ability to handle a large amount of data, which can directly find a good random source assigned to the doctor’s public–private key, and the whole system stores all the data, and there is no trustworthy third party involved, so that there is no need to worry about privacy leakage. Then verified by experiments, the results show that the algorithm in this paper can effectively verify the roles that need to apply for additions, deletions, and checks to ensure security. The limitation of this system lies in its popularity due to the relatively complex operating environment and lack of integrated configuration files, making it temporarily difficult to operate. From a system perspective, the underlying principles of expanding the system are all based on Ethereum, so some functions are difficult to implement without being constrained by Ethereum, such as the time of smart contract on chain, and so on. At the same time, the system has a mechanism that needs to be improved or perfected. If NFTs are added, it will greatly save time in verifying roles, but how to use them as credentials remains to be experimented with. For future work, our team will consider whether the verification process is faster and more efficient if we utilize the NFT^38^ participation verification approach. At the same time, smart contracts are more emerging than blockchain, the standard is still not perfect, and the mainstream is now mainly dominated by the Ethereum standard, so the arithmetic problem of ZK and some other minor problems still exist, and there are still metrics and techniques that have not yet been evaluated outside of the experiments. Finally, a model for integrating blockchain technology with machine learning and artificial intelligence (AI) solutions will ensure that our healthcare data management system can interact in a highly confidential and almost completely private environment, laying a solid foundation for personalized healthcare solutions and management.

## Data Availability

Data sets generated during the current study are available from the corresponding author on reasonable request. The data are available from Xing Zhang (zhang_xing@lnut.edu.cn). But restrictions apply to the availability of these data, which were used under license for the current study, and so are not publicly available.
